# Compression of the Rectum, Bladder, and External Iliac Vein Due to Hip Arthroplasty-Related Pseudotumor

**DOI:** 10.7759/cureus.20671

**Published:** 2021-12-24

**Authors:** Lucas D Winter, Kathryn C Helmig, Paul J Goodwyn, Rick J Gehlert

**Affiliations:** 1 Department of Orthopaedic Surgery and Rehabilitation, University of New Mexico School of Medicine, Albuquerque, USA; 2 Department of Orthopaedic and Traumatology, Sonoran Orthopaedic Trauma Surgeons, Scottsdale, USA

**Keywords:** total joint replacement, osteolysis, iliac vein, total hip arthroplasty, inflammatory pseudotumor

## Abstract

A 58-year-old male who underwent left total hip arthroplasty in 1988 for post-traumatic arthritis after an operatively-treated acetabular fracture presented with progressive left hip pain, lower extremity swelling, urinary urgency, constipation, and tenesmus (the feeling of needing to pass stool despite having an empty colon). Imaging demonstrated massive pseudotumor causing iliac vein compression and significant displacement of the rectum and bladder requiring decompression in combination with general surgery followed by revision hip arthroplasty four months later. This case highlights a unique constellation of symptoms due to pseudotumor after total hip arthroplasty, as well as the severity to which pseudotumor can progress, requiring staged decompression with general surgery before revision.

## Introduction

Pseudotumor occurs in the setting of inflammation due to total hip arthroplasty (THA) wear debris [1,2]. These are non-infectious and non-neoplastic solid or cystic inflammatory masses formed as a reaction to wear debris by macrophages and lymphocytes that cause local inflammation and necrosis of both soft tissue and bone [1,2]. Metal, polyethylene, ceramic, and polymethylmethacrylate have been implicated in the formation of pseudotumors, with metallic wear in the setting of metal-on-metal implants being the most common etiology [2]. Pseudotumor masses typically present with hip pain and gait disturbance but can also cause intrapelvic lesions with compression of local soft tissue structures. Previous case reports on lower extremity swelling and deep vein thrombosis (DVT) as a result of venous compression, urinary symptoms, and hydronephrosis, as well as vague abdominal fullness and groin pain, have been published in the literature [3-8]. Additionally, femoral and sciatic nerve palsies have been reported due to compression or encasement by pseudotumor [9]. Bone and soft tissue can also ultimately be affected and destroyed, leading to THA instability [10].

To our knowledge, compression of the rectum causing constipation and tenesmus due to pseudotumor after THA has not been documented in the literature. This case report details a complex case of pseudotumor 27 years after THA, which required coordination with general surgery for multiple abdominal surgeries followed by THA revision four months later.

The patient was informed that data concerning the case would be submitted for publication, and he provided informed consent for publication.

## Case presentation

**Figure 1:**
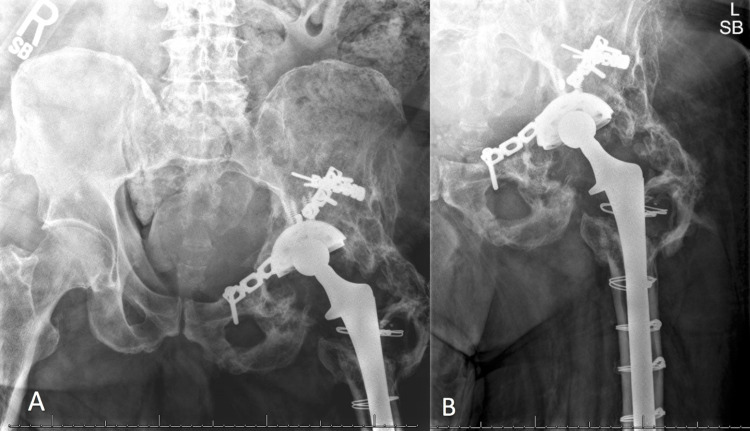
Preoperative radiographs of the pelvis. Preoperative anterior-posterior radiograph of the pelvis (a) and anterior-posterior radiograph of the left hip (b) demonstrating acetabulum hardware with broken plate, prior left total hip arthroplasty, heterotopic ossification, osteolysis, and large colonic stool burden.

A 58-year-old male presented to our clinic in May 2015 with worsening left hip pain, urinary urgency, constipation, and tenesmus in the setting of prior left metal-on-polyethylene THA in 1988 for post-traumatic arthritis after an operatively treated acetabular fracture sustained in 1986. The patient had done well after his total hip arthroplasty until March 2013, when he began having progressively worsening left hip pain, followed by intermittent left leg swelling, urinary urgency, constipation, and tenesmus. The patient had undergone multiple workups at outside hospitals, including hip aspirations, MRI, CT scan, and X-rays. X-rays demonstrated prior left acetabulum fixation with broken plate along the superior pubic ramus, heterotopic ossification, and prior left THA with large lytic lesions consistent with osteolysis (Figure 1).

**Figure 2:**
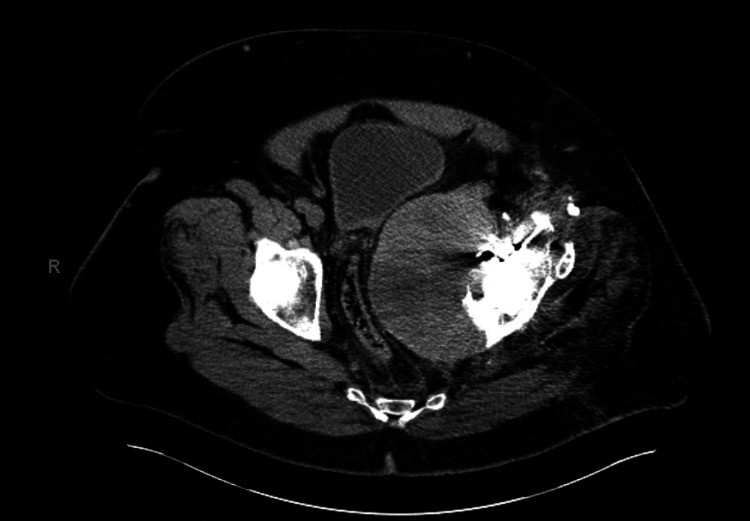
Preoperative CT scan. Preoperative axial CT scan demonstrating pseudotumor associated with left THA causing displacement of the bladder and significant narrowing of the rectum due to compression from the mass.

MRI and CT scan demonstrated a 13-centimeter heterogeneous cystic mass within the pelvis, closely associated with prior THA, thought to represent pseudotumor associated with osteolysis (Figure 2).

**Figure 3:**
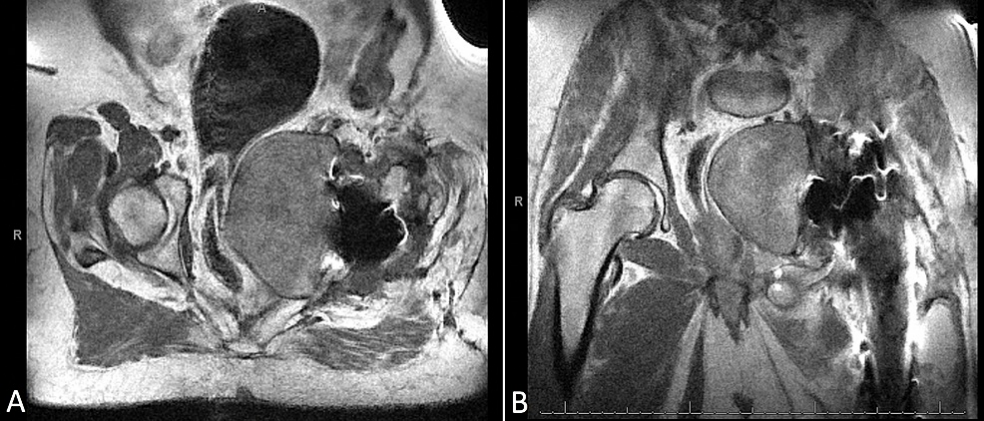
Preoperative MRI images. Preoperative axial MRI (a) demonstrating displacement of the bladder as well as compression on the rectum due to large pseudotumor associated with the left hip. Coronal MRI (b) demonstrating large cystic mass displacing iliac vessels and the rectum.

The mass was noted to be causing near-total iliac vein occlusion as well as displacement of the bladder and compression of the rectum (Figure 3).

Multiple left hip aspirations along with serum erythrocyte sedimentation rate (ESR) and C-reactive protein (CRP) were normal. The initial surgical plan included revision of the total hip arthroplasty with possible second surgery by general surgery for decompression of the abdominal mass if needed. 

The patient subsequently developed progressively worsening left lower extremity swelling and was referred to the general surgery clinic before revision THA. He underwent a lower extremity arterial and venous duplex ultrasound that was negative for thrombosis. At his appointment with general surgery in October 2015, he was noted to have discoloration of the left ankle and foot due to venous stasis secondary to left external iliac vein occlusion. His case was discussed with the orthopedics, general surgery, and vascular surgery services, and a plan for decompression and staged hip revision was developed. 

**Figure 4:**
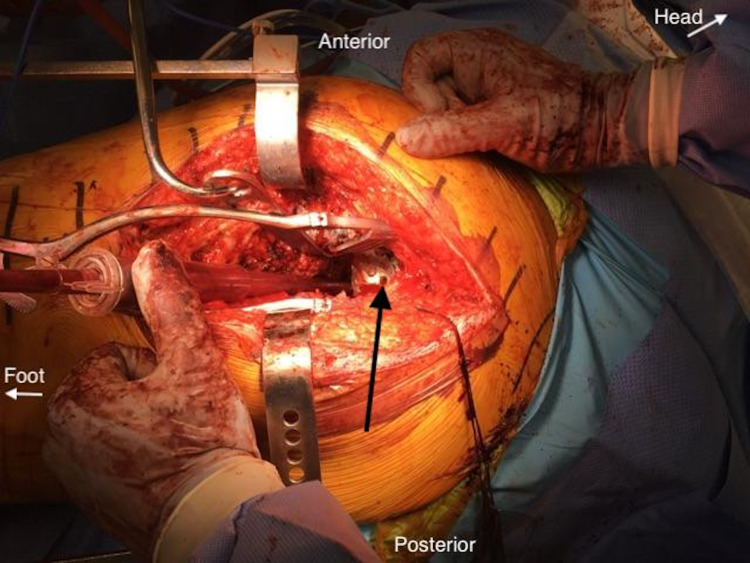
Intraoperative photograph during initial debridement. Intraoperative photograph demonstrating location of the preexisting quadrilateral surface defect which communicated with the intra-pelvic cyst (black arrow).

In November 2015, he underwent a combined procedure with orthopedics and general surgery for exploratory laparotomy, debulking, and decompression of the retroperitoneal mass. A standard laparotomy incision was utilized. Intraoperatively, the mass was noted to be significantly displacing the rectum and iliac vessels to the right. A screw and broken plate were removed from the left superior pubic ramus. The large cyst was then entered from a retroperitoneal extension through a preexisting defect in the quadrilateral surface (Figure 4).

**Figure 5:**
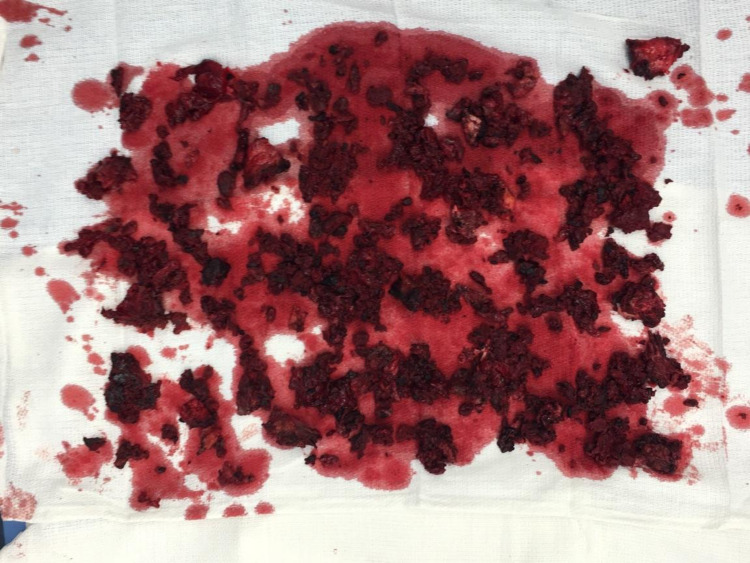
Intraoperative photograph of pseudotumor contents. Intraoperative photograph demonstrating clay-like contents/consistency of the pseudotumor.

A portion of the cyst wall was excised, and intra-abdominal and intra-cystic irrigation and debridement were performed. The mass contained a large volume of wear debris with a clay-like consistency (Figure 5).

Samples were sent for histopathology, which demonstrated a non-specific inflammatory reaction. Complete intraperitoneal excision was not undertaken due to the significant risk of adhesions associated with this approach. Two drains were placed, and the abdomen was closed with retention sutures and supra-fascial wound vac. 

**Figure 6:**
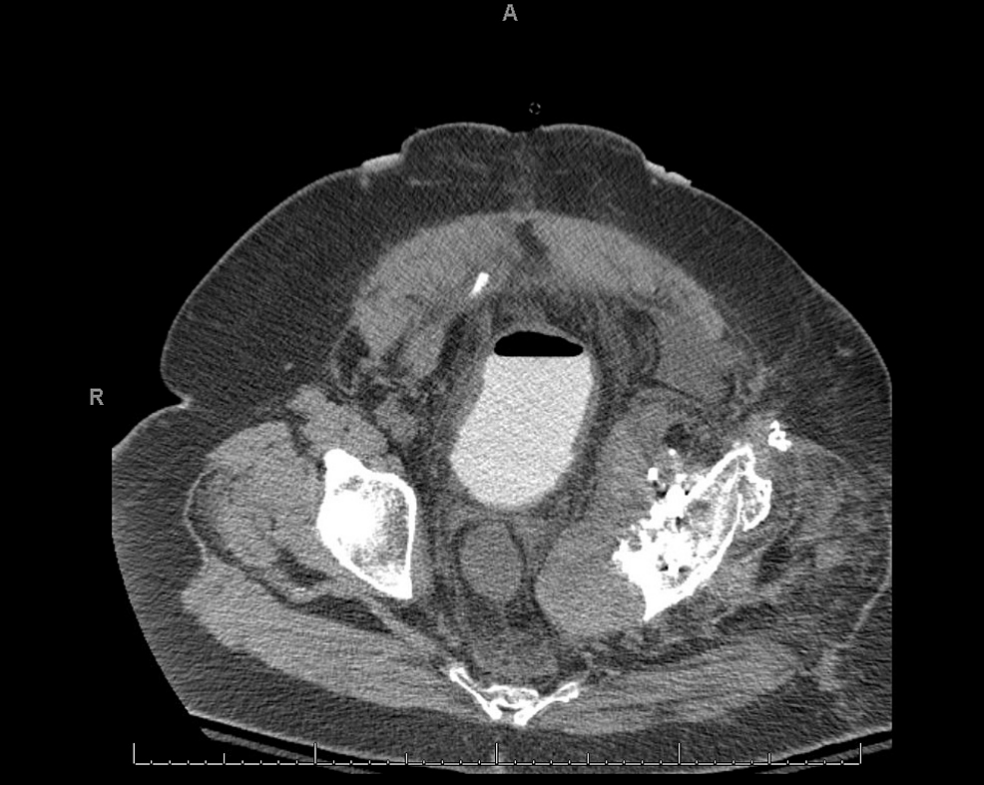
Postoperative CT scan. Postoperative axial CT scan demonstrating decompression of pseudotumor, with now normal-appearing location of the bladder and rectum without narrowing as seen preoperatively.

The patient returned to the operating room the following day with general surgery after developing increasing left lower quadrant abdominal pain and increased sanguineous output from the drains and wound vac. A CT scan was obtained before he was taken to the operating room, which was unremarkable aside from the notable decompression of the pseudotumor (Figure 6).

Intraoperatively he was noted to have significant pelvic bleeding and received six units of packed red blood cells. His abdomen and pelvis were explored, and the bleeding was believed to be associated with the left anterior branch of the obturator artery. This branch was unable to be found intraoperatively, therefore the pelvis was packed with lap pads and temporarily closed with ABThera open abdomen negative pressure therapy [KCI, San Antonio TX]. The patient was taken for angiography and underwent embolization of the anterior division of the left internal iliac artery and placement of an inferior vena cava (IVC) filter. He subsequently stabilized and did not require further blood transfusions. He returned to the operating room the following day for removal of pelvic packing and secondary abdominal closure with retention sutures and supra-fascial wound vac. He was discharged from the hospital with the supra-fascial wound vac, which was maintained and changed every 3-5 days until secondary healing was complete. In December 2015, the patient’s incision had completely healed, and he was noted to be voiding normally and having normal, consistent bowel movements with a resolution of his pre-operative symptoms. 

In March 2016, he underwent left THA revision consisting of head and liner exchange through the central aspect of the prior Kocher-Langenbeck incision. His prior acetabular shell and femoral stem were noted to have excellent fixation without loosening. The size 28 head and polyethylene liner were removed, and significant wear was noted centrally in the polyethylene liner. The trunnion was inspected, and no evidence of trunnionosis was present. Extensive scar tissue was noted around the joint and required debridement. Clay-like wear debris, similar to that encountered in the initial surgery, was again noted and debulked. The most proximal cable from the prior proximal femur cerclage was removed. The acetabular screw holes were curetted, and the anteroinferior screw holes were noted to enter the cyst. Upon entry of the cyst, approximately one liter of coffee-colored fluid was evacuated. This fluid was culture-negative and associated with polyethylene wear debris. A new size 32 metal head and highly cross-linked polyethylene liner were placed following thorough debridement.

**Figure 7:**
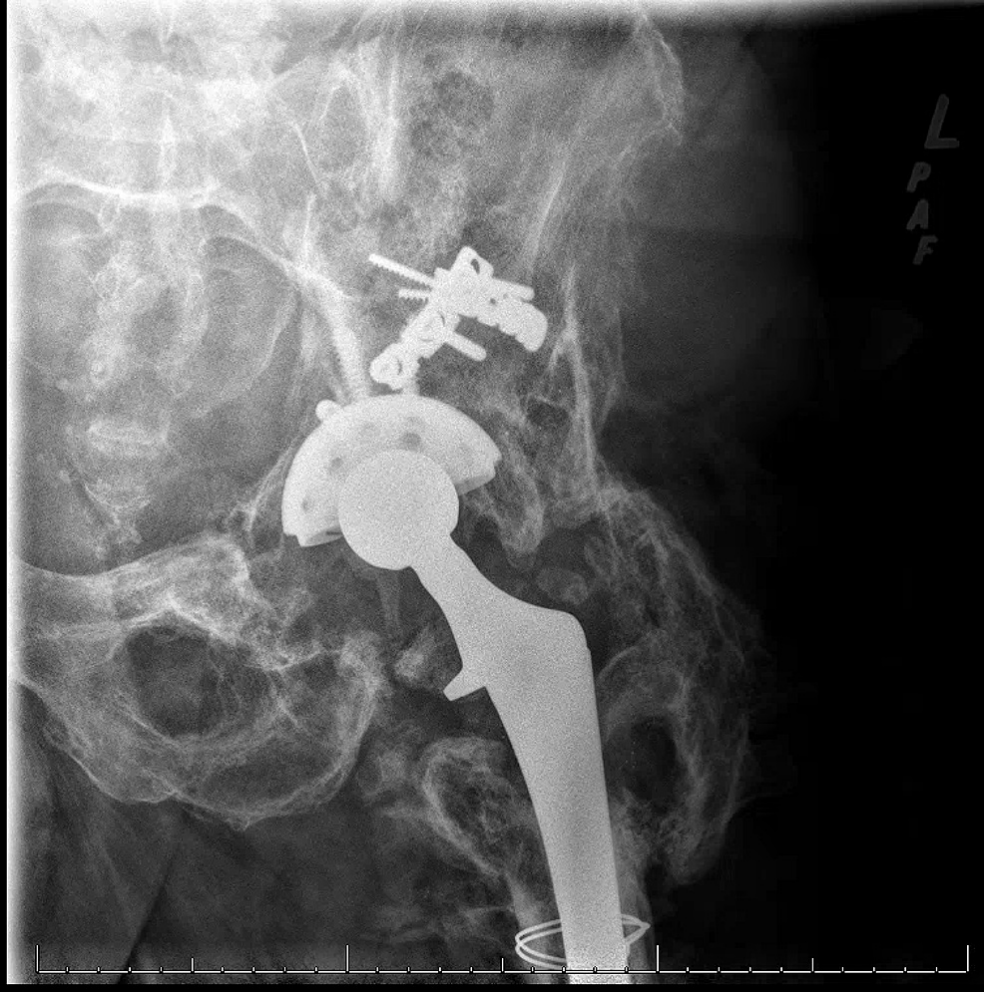
Postoperative radiographs. Postoperative anterior-posterior radiograph of the left hip demonstrating removal of broken acetabulum hardware and proximal cerclage wire with maintained left THA components in good alignment.

The patient’s most recent follow-up was in July 2020, and he was noted to have no left hip pain with the resolution of prior urinary and bowel symptoms. X-rays demonstrated left THA components in good alignment without evidence of ongoing osteolytic changes or hardware complications (Figure 7).

His IVC filter was removed in June 2016. He continues to have occasional, intermittent left lower extremity swelling, however not to the degree that was noted pre-operatively in 2015.

## Discussion

The case presented details, the course, and treatment of a large pseudotumor secondary to polyethylene acetabular liner wear with a unique constellation of presenting symptoms not previously described in the literature. In the case presented, several symptoms were present secondary to the mass effect. In some patients, pseudotumor may be asymptomatic. Patients with symptomatic pseudotumor typically undergo revision arthroplasty, while asymptomatic patients can be closely observed for clinical signs or symptoms as well as metal ion levels if metallic wear is suspected [1,2,10]. In general, if components are well-fixed, with no signs of damage to the locking mechanisms, in appropriate alignment with a large enough acetabular shell to accommodate the appropriate new femoral head size and the hip is stable intra-operatively, head and liner exchange is an option [1]. Head and liner exchange or revision of acetabular and/or femoral components is typically sufficient in the management of pseudotumors to prevent expansion [1,2,10]. However, debulking the mass may be necessary at the time of THA revision or before revision in the setting of severe symptoms from mass effect, as presented in our case. We recommend a discussion with general surgery, vascular surgery, urology, and/or gynecology before decompression if intrapelvic or intra-abdominal structures are being affected, particularly if the associated symptoms are the patient’s primary concern(s) at the time of presentation.

Multiple reports detail compression of the iliac vein secondary to pseudotumor, resulting in lower extremity swelling and/or deep vein thrombosis (DVT) [3-5]. A common aspect of treatment in patients with iliac vein compression, particularly in the setting of DVT, is the placement of an IVC filter [3-5]. The IVC filter is thought to reduce the risk of pulmonary emboli while handling the iliac vein during decompression [4]. In our case, an IVC filter was placed after the initial debulking surgery but before secondary abdominal surgery and ultimate THA revision. In the patient presented in our case, an additional indication for IVC filter was the anticipated need for further surgery and venous thromboembolism prophylaxis in a high-risk patient (due to open abdomen, involvement of the iliac vein, and active bleeding).

## Conclusions

Pseudotumor secondary to THA can present in a variety of ways, and as such, it is critical for clinicians in various specialties to be aware of this when evaluating patients with prior THA. Our case demonstrates a patient with a unique constellation of symptoms due to intrapelvic compression secondary to pseudotumor. The severity and complexity of these cases are noteworthy and often require a combined approach for treatment with orthopedics, general surgery, and vascular surgery, as in the case presented. The ultimate goal of treatment is to relieve compression on intra-abdominal or intrapelvic structures and ultimately perform THA revision to prevent worsening of the pseudotumor.
